# Supplementary material for: Past–future information bottleneck for sampling molecular reaction coordinate simultaneously with thermodynamics and kinetics
Source: Nat Commun. 2019 Aug 8;10:3573. doi: 10.1038/s41467-019-11405-4 (PMC6687748; doi:10.1038/s41467-019-11405-4)
Supplement: Supplementary file 1 — Supplementary Information [file 41467_2019_11405_MOESM1_ESM.pdf]

# Past–future information bottleneck for sampling molecular reaction coordinate simultaneously with thermodynamics and kinetics

**Wang et al.**

# Supplemental information to "Past–future information bottleneck framework for sampling molecular reaction coordinate, thermodynamics and kinetics"

## SUPPLEMENTARY NOTES

### Note 1

In this note we define various terms introduced in the main text as well. In all of these we use $P(\mathbf{X})$, $P(\mathbf{X},\mathbf{Y})$ respectively to denote the probability distribution of a random variable $X$ and the joint probability distribution of two random variables $\mathbf{X}$ and $\mathbf{Y}$. Other variables are the same as defined in the main text.

1. **Mutual information**

   This is a commonly used information theoretic measure to describe how much information is shared between two random variables $X$ and $Y$. It is defined as:

$$I(\mathbf{X},\mathbf{Y}) = \int P(\mathbf{X},\mathbf{Y}) \ln \frac{P(\mathbf{X},\mathbf{Y})}{P(\mathbf{X})P(\mathbf{Y})} d\mathbf{X} d\mathbf{Y} \tag{1}$$

2. **Shannon entropy**

   The Shannon entropy for a random variable $X$ is defined as:

$$H(\mathbf{X}) = -\int P(\mathbf{X}) \ln P(\mathbf{X}) d\mathbf{X} \tag{2}$$

3. **Cross entropy**

   The cross entropy between two probability distributions $P(\mathbf{X})$ and $Q(\mathbf{X})$ is given by:

$$C(\mathbf{X},\mathbf{Y}) = -\int P(\mathbf{X}) \ln Q(\mathbf{X}) d\mathbf{X} \tag{3}$$

4. **Kullback-Leibler Divergence**

   The Kullback-Leibler (KL) $D_{KL}(P||Q)$ divergence between two probability distributions $P(\mathbf{X})$ and $Q(\mathbf{X})$ is given by:

$$D_{KL}(P||Q) = \int P(\mathbf{X}) \ln \frac{P(\mathbf{X})}{Q(\mathbf{X})} d\mathbf{X} \tag{4}$$

5. **Exact decoder definition**

   The exact decoder can be defined as the the conditional probability distribution of $\mathbf{X}_{\Delta t}$ given a RC $\boldsymbol{\chi}$. It can be calculated by using Bayes theorem:

$$P(\mathbf{X}_{\Delta t}|\boldsymbol{\chi}) = \frac{P(\boldsymbol{\chi},\mathbf{X}_{\Delta t})}{P(\boldsymbol{\chi})} = \frac{\int P(\boldsymbol{\chi}|\mathbf{X})P(\mathbf{X},\mathbf{X}_{\Delta t})d\mathbf{X}}{\int\int P(\boldsymbol{\chi}|\mathbf{X})P(\mathbf{X},\mathbf{X'}_{\Delta t})d\mathbf{X}d\mathbf{X'}_{\Delta t}} \tag{5}$$

6. **Acceleration factor**

   As shown in [1], and under the conditions detailed there, it is possible recover the unbiased timescales from biased simulations through the calculation of a simple acceleration factor. If the biased simulation time is $t$, the associated unbiased timescale $\tau$ can be calculated by:

$$\tau = \int_0^t e^{\beta V[\boldsymbol{\chi}(t')]} dt' = \sum_{n=1}^{M} e^{\beta V^n} (t_n - t_{n-1}) \tag{6}$$

   where $V(\boldsymbol{\chi}(t'))$ is the bias experienced by the system at time $t'$, constructed as a function of the RC $\boldsymbol{\chi}$. This can equivalently be calculated as a discrete sum over the $M$ integration time-steps.

## Note 2

In this note we provide the missing details of various derivations given in the main text.

1. **Gibbs's inequality and variational lower bound**

   The bottleneck function $\mathcal{L}$ defined in the main text for our neural network architecture is given by a difference of two Shannon entropies[2]:

$$\mathcal{L} = I(\boldsymbol{\chi}, \mathbf{X}_{\Delta t}) = H(P(\mathbf{X}_{\Delta t})) - H(P_\theta(\mathbf{X}_{\Delta t}|\boldsymbol{\chi})) \tag{7}$$

   Gibbs's inequality[2] guarantees that that the KL-divergence between two probability distributions is always larger than 0. We thus have:

$$\begin{aligned} D_{KL}(P_{\boldsymbol{\theta}}(\mathbf{X}_{\Delta t}|\boldsymbol{\chi}))||Q_{\boldsymbol{\phi}}(\mathbf{X}_{\Delta t}|\boldsymbol{\chi})) &\equiv \int\int P_{\boldsymbol{\theta}}(\mathbf{X}_{\Delta t}, \boldsymbol{\chi}) \ln \frac{P_{\boldsymbol{\theta}}(\mathbf{X}_{\Delta t}|\boldsymbol{\chi})}{Q_{\boldsymbol{\phi}}(\mathbf{X}_{\Delta t}|\boldsymbol{\chi})} d\mathbf{X}_{\Delta t} d\boldsymbol{\chi} \\ &= \int\int P_{\boldsymbol{\theta}}(\mathbf{X}_{\Delta t}, \boldsymbol{\chi}) \ln P_{\boldsymbol{\theta}}(\mathbf{X}_{\Delta t}|\boldsymbol{\chi}) d\mathbf{X}_{\Delta t} d\boldsymbol{\chi} - \int\int P_{\boldsymbol{\theta}}(\mathbf{X}_{\Delta t}, \boldsymbol{\chi}) \ln Q_{\boldsymbol{\phi}}(\mathbf{X}_{\Delta t}|\boldsymbol{\chi}) d\mathbf{X}_{\Delta t} d\boldsymbol{\chi} \\ &= -H(P_{\boldsymbol{\theta}}(\mathbf{X}_{\Delta t}|\boldsymbol{\chi})) + C(P_{\boldsymbol{\theta}}(\mathbf{X}_{\Delta t}|\boldsymbol{\chi}), Q_{\boldsymbol{\phi}}(\mathbf{X}_{\Delta t}|\boldsymbol{\chi})) \end{aligned} \tag{8}$$

   Only in the limit that our approximate decoder is exactly the same as the exact inverse-Bayes decoder, $D_{KL}(P_\theta||Q_\phi)$ equals 0. By combining Eq.7 and Eq.8 , we get the relationship:

$$\mathcal{L} - D_{KL}(P_{\boldsymbol{\theta}}(\mathbf{X}_{\Delta t}|\boldsymbol{\chi}))||Q_{\boldsymbol{\phi}}(\mathbf{X}_{\Delta t}|\boldsymbol{\chi})) = H(P(\mathbf{X}_{\Delta t})) - C(P_{\boldsymbol{\theta}}(\mathbf{X}_{\Delta t}|\boldsymbol{\chi}), Q_{\boldsymbol{\phi}}(\mathbf{X}_{\Delta t}|\boldsymbol{\chi})) \equiv H(P(\mathbf{X}_{\Delta t})) + \mathcal{L}' \tag{9}$$

   Here, $H(P(\mathbf{X}_{\Delta t}))$ only depends on the data set and is independent of the parametrization of the encoder and the decoder. Thus the term $H(P(\mathbf{X}_{\Delta t})$ can be completely ignored while optimizing the parameters $\boldsymbol{\theta}$ and $\boldsymbol{\phi}$. Maximizing the objective function $\mathcal{L}' = -C(P_{\boldsymbol{\theta}}(\mathbf{X}_{\Delta t}|\boldsymbol{\chi}))$ is then equivalent to maximizing the lower bound of $\mathcal{L}$, which is the expression stated in Eq.4 in the main text.

2. **PIB objective $\mathcal{L}'$ for unbiased trajectory**

   For a unbiased trajectory $\{\mathbf{X}^1, \mathbf{X}^2, ..., \mathbf{X}^{M+k}\}$, for given $\boldsymbol{\theta}$, we can get corresponding $\{\boldsymbol{\chi}^1, \boldsymbol{\chi}^2, ..., \boldsymbol{\chi}^M\}$ using $\boldsymbol{\chi}_i = \sum_i c_i s_i$. We also have a sampling of the states of the system after corresponding $\Delta t$ intervals: $\{\mathbf{X}^{1+k}, \mathbf{X}^{2+k}, ..., \mathbf{X}^{M+k}\}$. Together the pair $(\boldsymbol{\chi}_i, \mathbf{X}_{i+k})$ sampled from the dataset follows the distribution $P_{\boldsymbol{\theta}}(\mathbf{X}_{\Delta t}|\boldsymbol{\chi})$. With this, we have:

$$\mathcal{L}' = -C(P_{\boldsymbol{\theta}}(\mathbf{X}_{\Delta t}|\boldsymbol{\chi}), Q_{\boldsymbol{\phi}}(\mathbf{X}_{\Delta t}|\boldsymbol{\chi})) = \int\int P_{\boldsymbol{\theta}}(\mathbf{X}_{\Delta t}, \boldsymbol{\chi}) \ln Q_{\boldsymbol{\phi}}(\mathbf{X}_{\Delta t}|\boldsymbol{\chi}) d\mathbf{X}_{\Delta t} d\boldsymbol{\chi} = \frac{1}{M} \sum_{n=1}^{M} \log\, Q(\mathbf{X}^{n+1}|\boldsymbol{\chi}^n) \tag{10}$$

3. **PIB objective $\mathcal{L}'$ for biased trajectory**

   For a biased trajectory $\{\mathbf{X}^1, \mathbf{X}^2, ..., \mathbf{X}^M\}$ with corresponding biasing potential values $\{V^1, V^2, ..., V^M\}$, the unbiased probability distribution of $\mathbf{X}$ can be calculated by:

$$P(\mathbf{X}) = \frac{\sum_i \delta(\mathbf{X}^i - \mathbf{X}) e^{\beta V^i}}{\sum_i e^{\beta V^i}} \tag{11}$$

   The encoder $P(\boldsymbol{\chi}|\mathbf{X})$ and decoder $P(\mathbf{X}_{\Delta t}|\boldsymbol{\chi})$ are taken to be independent of the bias. The first assumption is strictly true, while the second is valid for small enough $\Delta t$ as explained in the main text. Therefore

$$\mathcal{L}' = \int\int P_{\boldsymbol{\theta}}(\mathbf{X}_{\Delta t}|\boldsymbol{\chi}) P(\boldsymbol{\chi}) \ln Q_{\boldsymbol{\phi}}(\mathbf{X}_{\Delta t}|\boldsymbol{\chi}) d\mathbf{X}_{\Delta t} d\boldsymbol{\chi} = \left\{ \sum_{n=1}^{M} e^{\beta V^n} \right\}^{-1} \sum_{n=1}^{M} e^{\beta V^n} \log\, Q(\mathbf{X}^{n+1}|\boldsymbol{\chi}^n) \tag{12}$$

## Note 3

In this note we provide details of the mutual information calculation for critical residue prediction. We use the backbone dihedral angles to describe the motion of each residue. Here we denote them as $\phi_i, \psi_i$, where $i$ is the index

of a particular residue. We use $I(\theta, \chi)$ to quantify the correlation between dihedral angle $\theta$ (where $\theta$ could be $\phi$ or $\psi$) and the unbinding process, where $I(\theta, \chi)$ is mutual information between a dihedral angle $\theta$ and the RC $\chi$.

For each residue, we have two dihedral angles. At the same time, for one dihedral angle, we can calculate its mutual information with $\chi_1$ or $\chi_2$. So we have four quantities for each residue( $I(\phi, \chi_1)$,$I(\psi, \chi_1)$, $I(\phi, \chi_2)$ and $I(\psi, \chi_2)$ ). We rank the importance of each residue by the maximum of the four quantities. Here we only consider the parts of trajectory that is bias-free($\chi_1 > 0.4$ and $\chi_2 > 0.3$). Because we require that the energy barriers between metastable states should be bias-free to ensure we can get the correct reweighted dynamics of the unbinding process. However, we only guarantee that the main energy barrier between bound and unbound state has zero bias when we perform biased MD simulation while bias can still be added on barriers between other metastable states. Looking at the unbiased region all us to reduce the influence of the biasing potential on the dynamics of the system and focus more on the transition states.

## SUPPLEMENTARY METHODS

1. **Simulation set-up for Alanine dipeptide in vacuum**

   We follow [3] to set up our simulation for alanine dipeptide in vacuum. The simulations are performed with the software GROMACS 2016/GROMACS 5.0[4, 5], patched with PLUMED 2.4[6]. We constrain bonds involving hydrogens using the LINCS algorithm[7] and employ an integration time-step of 0.002 ps. The temperature is kept constant at 300K using the velocity rescaling thermostat (relaxation time of 0.1 fs)[8]. We employ no periodic boundary conditions and no cut-offs for the electrostatic and non-bonded Van der Waals interactions.

2. **Simulation set-up for L99A T4L-benzene**

   We follow [9] to set up our simulation for L99A T4L-benzene. The simulations are performed with the software GROMACS 2016/GROMACS 5.0[4, 5] patched with PLUMED 2.4[6]. The simulations are done with the constant number, pressure, temperature (NPT) ensemble with temperature 298 K and pressure 1.0 bar. Constant pressure is maintained using Parrinello-Rahaman barostat while the constant temperature is maintained using the v-rescale thermostat (modified Berendsen thermostat)[10]. The simulation box with periodic boundary condition is filled with TIP3P water. The side lengths of the box are $10\mathring{A}$ and there are around $10,000$ water molecules. The interaction is described by the force field CHARMM22*. The integration time step here as well was taken to be 2 fs.

## SUPPLEMENTARY DISCUSSION

To discuss the flexibility in choosing basis functions, we apply the same framework with another set of order parameters. As shown in Supplementary Table 1 and Supplementary Table 2, there are two central differences between this new set of order parameters and the set we discussed in the main text: (a) all helix-helix distances have been removed, (b) protein-ligand distances are defined by the distance between ligand and a new set of residues. In Supplementary Figure 6(a), the alpha carbons used to defined the protein-ligand distances in two basis function sets are colored differently, and our results demonstrate that the choices of distances are fairly arbitrary.We followed the protocol discussed in the main text. Only two minor changes were made, demonstrating further robustness of our protocol. First, we extended the simulation time from 4ns to 10ns in each trial. Second, the energy was increased by 8kJ in each round instead of 5kJ. We should stress that these two parameters can be chosen from a relatively wide range as long as the simulation time is long enough to sample local configurations and the increment of bias is not too big to include the poorly sampled region.

Order parameters weights in each RC are shown in Supplementary Figure 6 (b). With these, we were able to gain unbinding trajectories to do the critical residues calculation as discussed before. Since RCs were constructed from a basis function set that can't fully describe the whole system, they only contained partial information of the unbinding process. With this new set of basis function, critical residue calculation does not give exactly the same ranking as shown in the main text, however, as shown in Supplementary Figure 6 (c), the key residues are preserved. Some of the important residues are: Phe114, Gly110, Val111, Ala112, Thr109, Ile29, Glu108, Ser136, Lys135 and Trp138. These residues can still be classified into two broad groups: (a) residue 114, residue 135, residue 136, residue 138 and residues 108–112 contribute to breathing movement (b) residue 29 lies in a disordered region and can be picked due to noise or existence of likely allosteric communication pathway. These results show that the method is robust in terms of the choice of order parameters and has the potential to be applied to studying other complex systems.

## SUPPLEMENTARY FIGURES

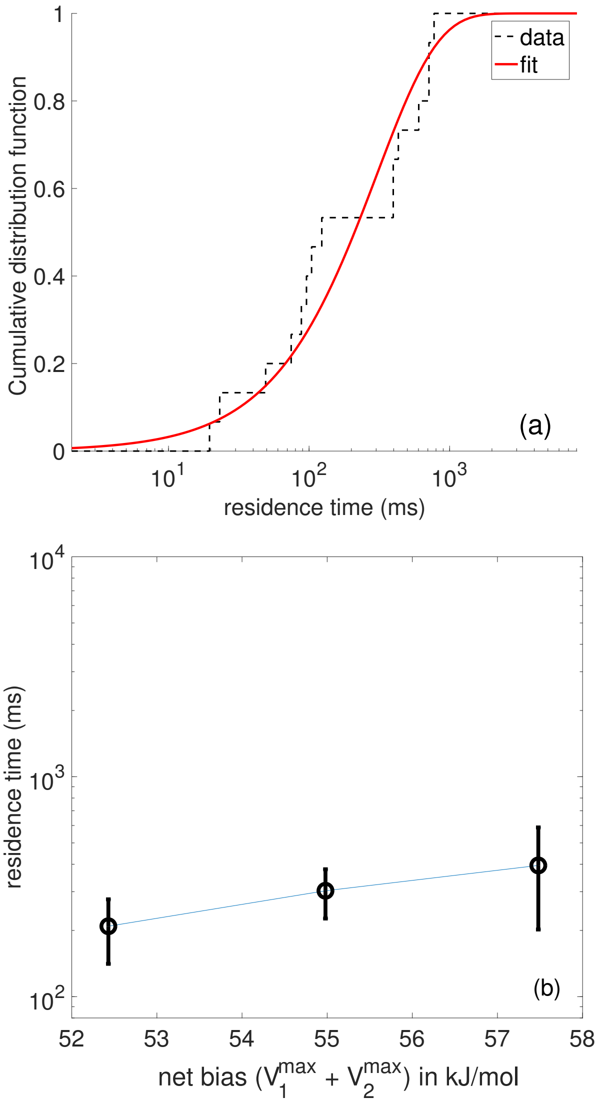


**Supplementary Figure 1:** (a) Poisson fit as per Supplementary Reference [11] to the 15 independent transition times obtained for the benzene-lysozyme dissociation constant with net maximum bias $V_1^{max} + V_2^{max} = 52$ kJ $\cdot$ mol$^{-1}$. The $k_{off}$ reported in the main text is calculated as the inverse of the fitted time constant here, $303 \pm 76$ ms. In (b), we provide the fitted time constant with associated error bars for 3 different biasing protocols, demonstrating convergence of the estimated time constant, at least on a log-scale. It is worth noting that for the weakest bias, we had the lowest number of dissociation events and hence the time-constant is a lower bound due to not having captured enough slow events. Hence the agreement should in principle be even better.

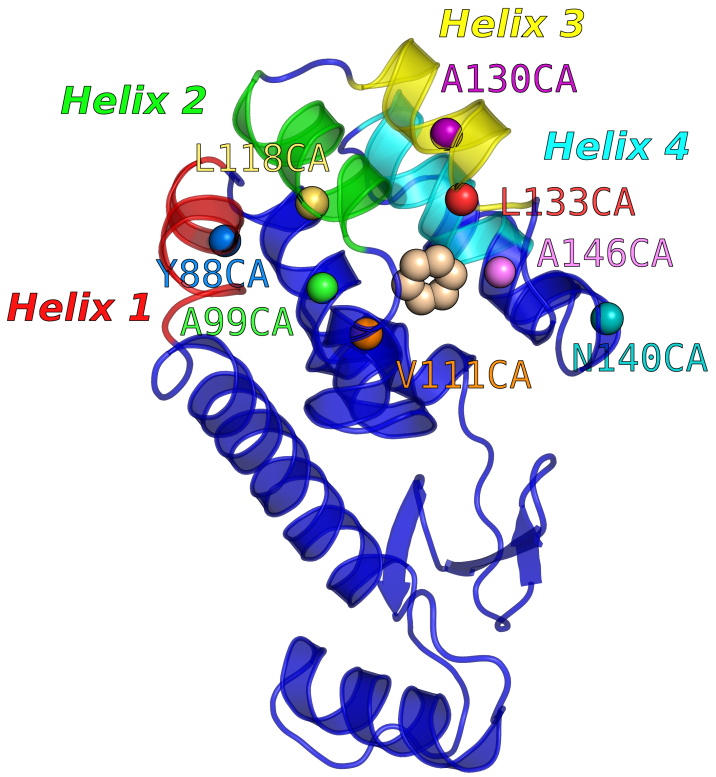


**Supplementary Figure 2:** Definition of Order parameters for Benzene-Lysozyme dissociation: Our 11 order parameters comprise 8 protein-ligand contacts and 3 protein-protein contacts. The protein-ligand contacts are implemented through the distances between the centres-of-mass of ligand to different protein atoms labeled in the plot. The protein-protein contacts are implemented through the distance between the centres-of-mass of ligand to centres-o-mass of helices l abeled in the plot. Further details are provided in Supplementary Table 1.

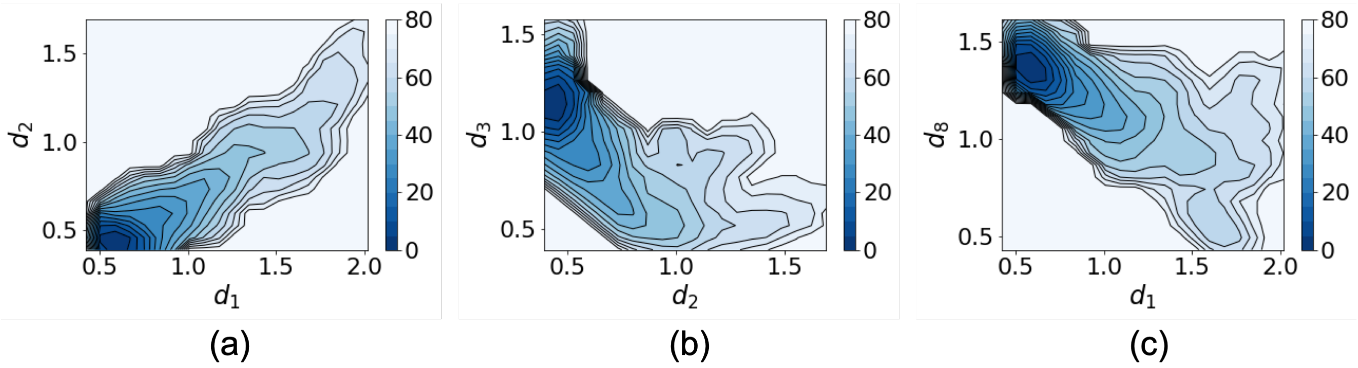


**Supplementary Figure 3:** Representative selected free energy surfaces for Benzene-Lysozyme dissociation obtained from our approach: (a) Free energy along $d_1$ and $d_2$. (b) Free energy along $d_2$ and $d_3$. (c) Free energy along $d_1$ and $d_8$. All energies are in units of $\mathrm{kJ} \cdot \mathrm{mol}^{-1}$ with contours every 5 $\mathrm{kJ} \cdot \mathrm{mol}^{-1}$. These surfaces are in excellent agreement with previous benchmarks on this system [9].

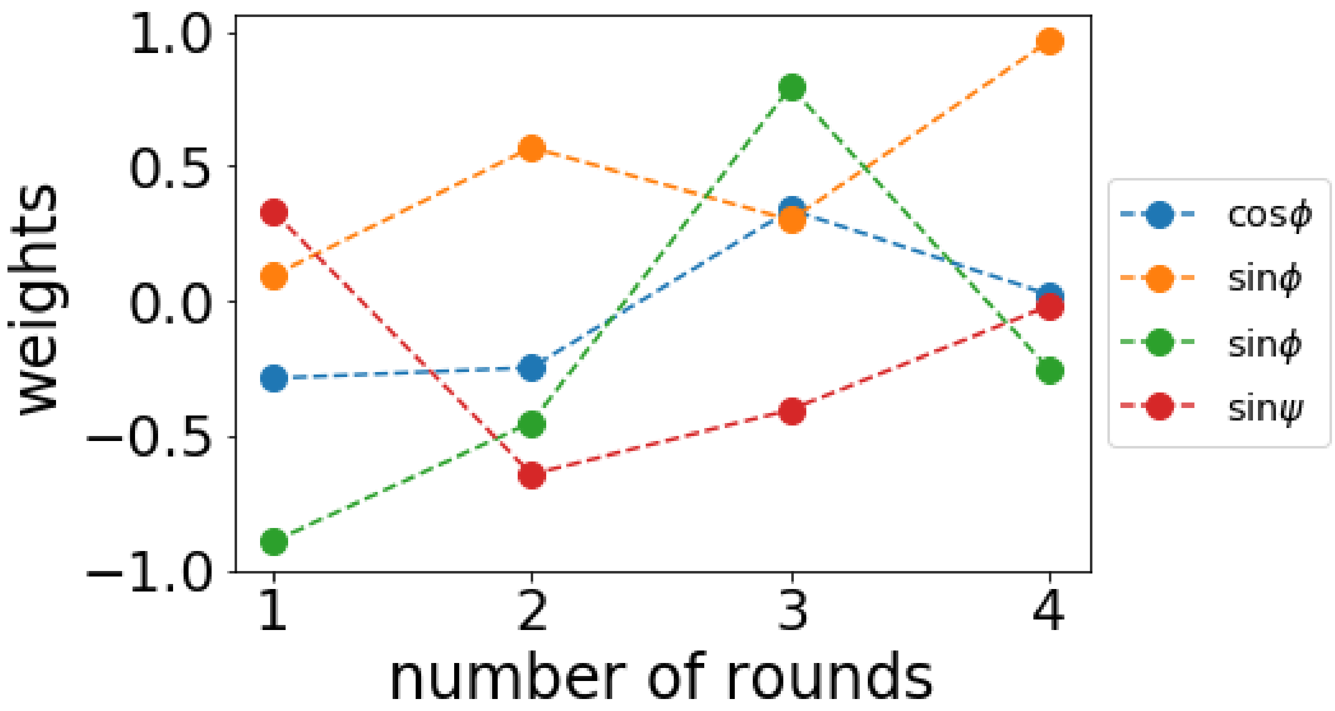


**Supplementary Figure 4:** Alanine dipeptide in vacuum: The value of weights for 4 order parameters a function of number of rounds.

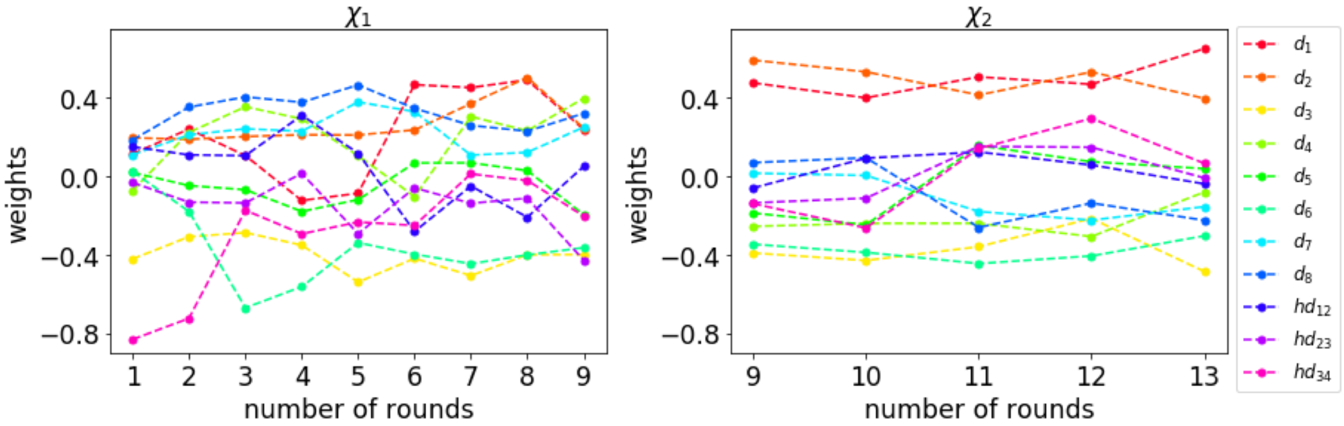


**Supplementary Figure 5:** Benzene-lysozyme dissociation: The plot on left shows the value of weights for 11 order parameters in $\chi_1$ as a function of number of rounds. The plot on right shows the value of weights for 11 order parameters in $\chi_2$ as a function of number of rounds. The plot on the right begins at 9 rounds directly because $\chi_2$ was not trained until 8 rounds.

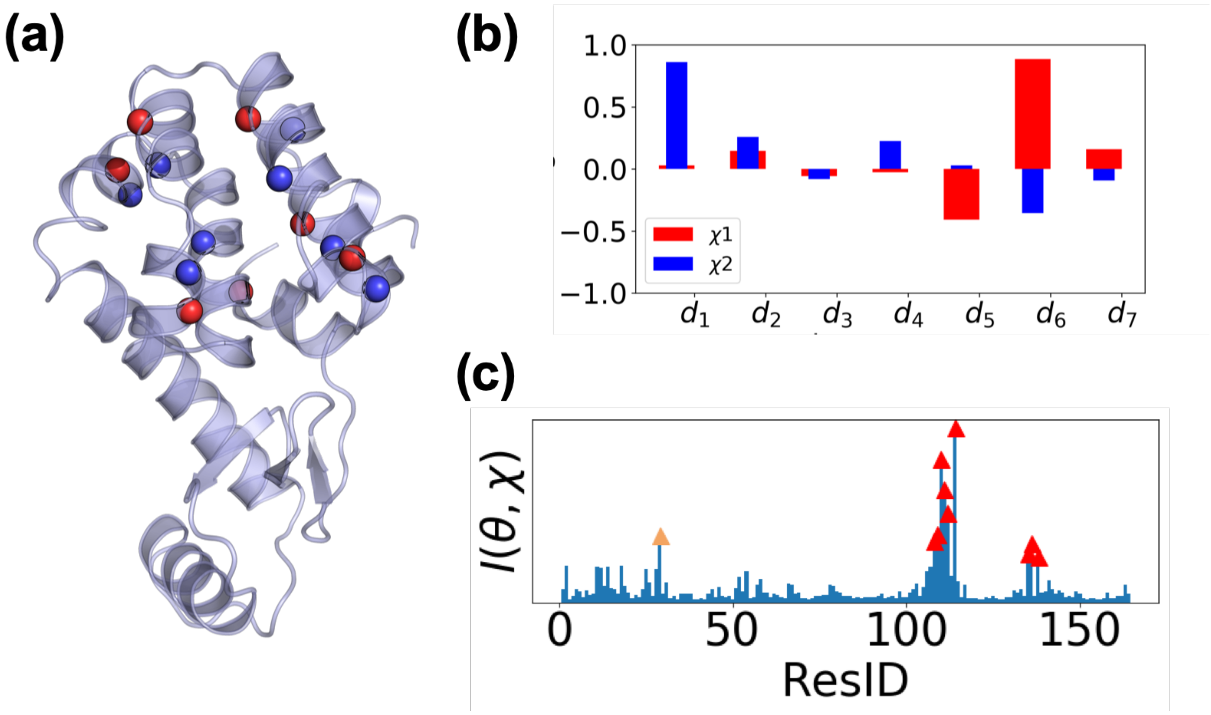


**Supplementary Figure 6:** (a) Alpha carbons used to define two sets of basis functions. Carbon atoms used in main text are colored in blue and the carbon atoms used in **Supplementary Discussion** are colored in red. (b) The 2-component Predictive Information Bottleneck for benzene-lysozyme dissociation. Order parameters are defined by Supplementary Table 2. (c) Critical residue analysis from new set of order parameters defined by Supplementary Table 2

**SUPPLEMENTARY TABLES**

| **Order parameter** | **Type** | **Definition** | **Weight $c_i$ in optimized $\chi_1 = \sum c_i s_i$** | **Weight in optimized $\chi_2 = \sum c_i s_i$** |
|---|---|---|---|---|
| $d_1$ | Protein-ligand | Y88CA–ligand | 0.4930 | 0.6524 |
| $d_2$ | Protein-ligand | A99CA–ligand | 0.5059 | 0.3984 |
| $d_3$ | Protein-ligand | L133CA–ligand | -0.4002 | -0.4853 |
| $d_4$ | Protein-ligand | L118CA–ligand | 0.2335 | -0.0792 |
| $d_5$ | Protein-ligand | V111CA–ligand | 0.0300 | 0.0399 |
| $d_6$ | Protein-ligand | A130CA–ligand | -0.4009 | -0.3037 |
| $d_7$ | Protein-ligand | N140CA–ligand | 0.1229 | -0.1544 |
| $d_8$ | Protein-ligand | A146CA–ligand | 0.2313 | -0.2240 |
| $hd_{12}$ | Protein-protein | Helix 1 (A82-S90) – Helix 2 (T115-123Q) | -0.2081 | -0.0378 |
| $hd_{23}$ | Protein-protein | Helix 2 (T115-123Q) – Helix 3 (W126-A134) | -0.1114 | -0.0073 |
| $hd_{34}$ | Protein-protein | Helix 3 (W126-A134) – Helix 4 (K147-T155) | -0.0204 | 0.0670 |

Supplementary Table 1: List of order parameters used to construct RC in the main text and their weights in $\chi_1$ and $\chi_2$.

| Order parameter | Type | Definition | Weight $c_i$ in optimized $\chi_1 = \sum c_i s_i$ | Weight in optimized $\chi_2 = \sum c_i s_i$ |
|---|---|---|---|---|
| $d_1$ | Protein-ligand | V87CA–ligand | 0.0297 | 0.8604 |
| $d_2$ | Protein-ligand | N101CA–ligand | 0.1448 | 0.2590 |
| $d_3$ | Protein-ligand | G110CA–ligand | -0.0583 | -0.0813 |
| $d_4$ | Protein-ligand | A119CA–ligand | -0.0261 | 0.2256 |
| $d_5$ | Protein-ligand | A129CA–ligand | -0.4076 | 0.0282 |
| $d_6$ | Protein-ligand | Y139CA–ligand | 0.8846 | -0.3548 |
| $d_7$ | Protein-ligand | V149CA–ligand | 0.1593 | -0.0921 |

Supplementary Table 2: List of order parameters used to construct RC in **Supplementary Discussion** and their weights in $\chi_1$ and $\chi_2$.

## SUPPLEMENTARY REFERENCES


[1] P. Tiwary and M. Parrinello, Phys. Rev. Lett. **111**, 230602 (2013).
[2] D. J. MacKay and D. J. Mac Kay, Information theory, inference and learning algorithms (Cambridge university press, 2003).
[3] O. Valsson and M. Parrinello, Phys. Rev. Lett. **113**, 090601 (2014).
[4] H. Berendsen, D. van der Spoel, and R. van Drunen, Computer Physics Communications **91**, 43 (1995).
[5] M. J. Abraham, T. Murtola, R. Schulz, S. Pll, J. C. Smith, B. Hess, and E. Lindahl, SoftwareX **1-2**, 19 (2015).
[6] G. A. Tribello, M. Bonomi, D. Branduardi, C. Camilloni, and G. Bussi, Comp. Phys. Comm. **185**, 604 (2014).
[7] B. Hess, H. Bekker, H. J. C. Berendsen, and J. G. E. M. Fraaije, Journal of Computational Chemistry **18**, 1463 (1997).
[8] G. Bussi, D. Donadio, and M. Parrinello, The Journal of Chemical Physics **126**, 014101 (2007), https://doi.org/10.1063/1.2408420.
[9] J. M. L. Ribeiro and P. Tiwary, Journal of chemical theory and computation (2019), 10.1021/acs.jctc.8b00869.
[10] G. Bussi, D. Donadio, and M. Parrinello, J Chem Phys **126**, 014101 (2007).
[11] M. Salvalaglio, P. Tiwary, and M. Parrinello, J. Chem. Theor. Comp. **10**, 1420 (2014).
